# Water, sanitation and hygiene risk factors associated with diarrhoea morbidity in a rural community of Enugu, South East Nigeria

**DOI:** 10.11604/pamj.2020.37.115.17735

**Published:** 2020-10-02

**Authors:** Ugochukwu Uzoechina Nwokoro, Onyekachi Ugwa, Chinemerem Daniel Onwuliri, Izuchukwu Frank Obi, Murphy-Okpala Ngozi, Chuka Agunwa

**Affiliations:** 1 Department of Community Medicine, University of Nigeria Teaching Hospital, Enugu, Nigeria,; 2 Nigeria Field Epidemiology and Laboratory Training Program, Abuja, Nigeria

**Keywords:** Determinants, diarrhoea, environmental factors, hand washing, community-based

## Abstract

**Introduction:**

diarrhoea remains a public health problem globally with majority of diarrhoea morbidity and mortality occurring in low resource settings. This study assessed the prevalence of diarrhoea and factors associated with diarrhoea in a rural community in Enugu, South East Nigeria.

**Methods:**

a community-based cross-sectional survey was conducted between May and June, 2017. Information on socio-demographic characteristics, water, sanitation, hand washing practices and diarrhoea history was obtained from 534 community residents using a structured interviewer administered questionnaire. Data were analyzed using descriptive statistics, Chi-square and logistic regression tests at 5% level of significance.

**Results:**

prevalence of diarrhoea in the two weeks preceding the study was 7.47% and 10.77% among all ages and children younger than five years respectively. Of 469 residents aged five years and above, 206 (43.92%) accessed source of drinking water within 30 minutes round trip walking distance from their households, 275 (58.64%) practiced open defecation while 456 (97.23%) and 455 (97.01%) reported washing hands with soap or ash and water before eating and after using the toilet respectively. Two or more households sharing a toilet facility [AOR = 4.78 (95% CI 2.03-11.24)] was a risk factor for diarrhoea while washing hands with soap or ash and water before eating [AOR = 0.23 (95% CI 0.06-0.90)] and after using the toilet [0.16 (95% CI 0.04-0.55)] protected against diarrhoea.

**Conclusion:**

increasing access to improved sanitary sewage disposal methods and promoting hand washing with soap and water at critical moments would improve diarrhoeal disease control.

## Introduction

Diarrhoea remains a public health problem globally despite efforts towards its control. In 2016, there were an estimated 4.5 billion episodes of diarrhoea worldwide. Diarrhoea ranked eight among the leading causes of mortality globally, accounting for over 1.6 million deaths among all ages and fifth leading cause of death among children younger than five years with 446,000 deaths. Approximately 90% of these diarrhoeal deaths occurred in South Asia and sub-Saharan Africa [1].

Globally, there have been significant reduction in morbidity and mortality due to diarrhoea in all ages including children younger than five years of age [2-4], however, 58% of diarrhoeal deaths still occur in Low and Middle Income Countries (LMICs) due to inadequate water, sanitation and hygiene [5, 6]. In sub-Saharan Africa, there are over one billion diarrhoeal episodes and an estimated 606,024 diarrhoeal deaths annually with nearly half of the deaths occurring in children younger than five years. Malnutrition, unsafe water and unsafe sanitation are the leading risk factors for dairrhoeal mortality among children younger than five years while unsafe water and unsafe sanitation are the leading risk factors for diarrhoeal mortality among all ages [1]. Furthermore, unsafe water and sanitation as well as other risks associated with poverty and poor development have been projected to be major contributors to wide gaps in health outcomes in the sub-Saharan Africa region by 2040, if current trends persist [7]. In Nigeria, there are an estimated 151,700 annual child deaths due to diarrhoea [8] with the prevalence of diarrhoea ranging between 10% and 18.8% [8, 9] and 80,968 deaths due to unsafe water, sanitation and hygiene [6] thus making Nigeria one of the leading contributors to diarrhoeal morbidity and mortality worldwide.

Although diarrhoeal cases occur globally among all regions and populations, majority of cases occur in low resource settings and in places where access to health care, safe water and sanitation are limited [3]. Understanding the contributions of water, sanitation and hygiene factors associated with diarrhoea in poor resource settings would help inform policy makers on areas where specific interventions could improve diarrhoeal disease control and contribute towards achieving the Sustainable Development Goal (SDG) 6 which aims to ensure access to water and sanitation for all by 2030. Although many rural communities often have inadequate access to safe water, sanitation and hygiene with socioeconomic, environmental and behavioural factors influencing diarrhoea occurrence [10], there is however paucity of data on the relative contribution of these potential risk factors to the burden of diarrhoeal disease in our study setting. The findings from this study would help guide policy formulation towards diarrhoea prevention and control in the study area. Thus, this study aimed to determine the prevalence of diarrhoea in a rural community in Enugu, South East Nigeria and to assess associated water, sanitation and hygiene risk factors of diarrhoeal disease.

## Methods

**Study area:** the study was conducted in Obukpa, a rural community in Nsukka Local Government Area of Enugu State, South East Nigeria. Obukpa has an estimated population of 60,000 and comprises four autonomous communities namely: Owerre, Obige, Ogbagu and Ajuona with a total of 57 villages. The study area is inhabited mostly by locals, majority of whom have no formal education or at most secondary level of education. They are engaged primarily in traditional occupations such as farming and petty trading. Most of the houses in the study area are built with mud and bricks with majority having no in-door piped water supply.

**Study design:** the study was a cross sectional community-based household survey conducted between May and June, 2017.

**Study population:** the study population comprised permanent residents of Obukpa community in Nsukka Local Government Area of Enugu State who had lived in the community for at least three months prior to the survey.

**Sample size determination and sampling procedure:** a minimum sample of 516 community residents was calculated using the Leslie Kish formula based on the assumption of two-week diarrhoea prevalence in Nigeria being 18.8% [8], design effect of 2, precision of 5%, and adjusting for a non-response rate of 10%. A two-stage cluster sampling technique was used to recruit study participants in which Stage one: one village was randomly selected by balloting from a frame of all the villages in each of the four autonomous communities in Obukpa, giving a total of four selected villages namely Ogwashi-Ujom, Obigeti-Akpogu, Aguohuru, and Amaeze-Uwani. Stage two: in each selected village, all households were enumerated and included in the study. Relevant information was obtained from all adult members of the households. Information on minors was obtained from the head of household or the spouse.

**Study instrument and data collection method:** a pre-tested structured interviewer administered questionnaire consisting of the following sections: socio-demographic data, water, sanitation and hand washing practices as well as history of diarrhoea in the two weeks preceding the survey was used to obtain information from community residents. Diarrhoea was defined as passage of loose or watery stool at least three times per day or more frequently than is normal for an individual [8] at any time within the two weeks prior to the survey. The questionnaire was prepared in English and translated to the local language, Igbo. To ensure the quality of data collection, a two-day training was done for four data collectors (trainee Community Health Officers and fluent in the local language) and two supervisors (junior resident doctors). The content of the training included the nature of the questionnaire, interview techniques, approach to household heads and each household member, how to double check filled questionnaires and data entry into an electronic database.

**Data analysis:** data were coded and statistical analysis conducted using Microsoft Excel and Epi info version 7.1.5.2 software. Frequencies and proportions were computed as descriptive statistics. At bivariate analysis, the association between diarrhoea and independent variables such as age, sex, educational status, source of drinking water, procurement of water, accessibility to source of drinking water, hand washing before eating and after visiting the toilet, sewage disposal method and sharing of toilet facility was determined using Chi-square or Fisher exact tests where appropriate with odds ratios and corresponding 95% confidence intervals computed and presented as measure of association. Logistic regression analysis was peformed to identify risk factors for diarrhoea (α = 5.0%).

**Ethical considerations:** the study was conducted as part of a regularly scheduled community intervention project of the Department of Community Medicine, University of Nigeria Teaching Hospital, Enugu in collaboration with local health authorities in Enugu State. Ethical approval for this study was obtained from the Health Research Ethics Committee of the University of Nigeria Teaching Hospital, Enugu. Permission to conduct the study was sought and obtained from the traditional leaders of the autonomous communities. Informed consent was obtained from all study participants after explaining the study and the participants showing full understanding of the study. Confidentiality of all the participants was assured and maintained during and after the study.

## Results

**Socio-demographic characteristics of community residents:** there were a total of 534 residents from 103 households in this study. Nearly half 266 (49.81%) of the residents were aged 25 years and above while 65 (12.17%) were children younger than 5 years. The median age (range) of children younger than 5 years was 24 (0-49) months while the median age (range) for those 5 years and above was 27 (5-98) years. Residents who had at most secondary level of education were 335 (62.73%) while 150 (28.09%) had no formal education (Table 1).

**Table 1:** socio-demographic characteristics of residents in selected communities, Obukpa, Enugu, South East Nigeria, May - June, 2017

Variable	Frequency	Proportion (%)
**Age (Years)**		
< 5	65	12.27
5- 9	54	10.11
10-24	149	27.90
≥ 25	266	49.81
**Sex**		
Male	218	40.82
Female	316	59.18
**Marital status**		
Single	311	58.24
Married	177	33.15
Divorced	2	0.37
Widowed	44	8.24
**Educational level**		
No formal education	150	28.09
Primary	145	27.15
Secondary	190	35.58
Tertiary	49	9.18
**Occupation**		
Farming	108	20.22
Artisan/petty trading	89	16.67
Government employee	33	6.18
Private company employee	24	4.49
Student	168	31.46
Unemployment	112	20.97

**Prevalence of diarrhoea:** in the two weeks preceding the survey, of the 534 residents, 40 had diarrhoea giving an overall diarrhoea prevalence of 7.49%, while among a total of 65 children younger than 5 years, 7 had diarrhoea with two-week diarrhoea prevalence of 10.77%.

**Water, sanitation and hygiene characteristics:** of the community residents aged five years and above, 417 (88.91%) reported borehole as the main source of drinking water while 41 (8.74%) reported rain water. Majority 349 (74.41%) of respondents buy drinking water from commercial borehole vendors. Less than half 206 (43.92%) of the residents accessed their source of drinking water within 30 minutes round trip walking distance from their households. Two hundred and seventy five (58.64%) of the residents practiced open defecation while 456 (97.23%) and 455 (97.01%) reported washing hands with soap or ash and water before eating and after using the toilet respectively (Table 2).

**Table 2:** water, sanitation and hygiene practices of residents in selected communities, Obukpa, Enugu, South East Nigeria, May - June, 2017

Variable	Frequency	Proportion (%)
**Source of drinking water**		
Borehole	417	88.91
Rain water	41	8.74
Public tap/pump	11	2.35
**Procurement of water**		
Commercially procured	349	74.41
Non-commercially procured	120	25.59
**Accessibility to source of drinking water (on foot)**		
≤ 30 minutes	206	43.92
> 30 minutes	263	56.08
**Sewage disposal method**		
Open defecation	275	58.64
Pit latrine	146	31.13
Pour flush	7	1.49
Water closet	41	8.74
**Hand washing before eating**		
Yes	456	97.23
No	13	2.77
**Hand washing after using toilet**		
Yes	455	97.01
No	14	2.99

**Factors associated with diarrhoeal disease:** Table 3 shows the crude and adjusted odds ratios for factors associated with diarrhoeal disease in the two weeks preceding the survey. At the bivariate level, socio-demographic characteristics such as age, sex and educational status; water and sanitation factors such as source of drinking water, whether water was commercially procured or otherwise and sewage disposal method were not significantly associated with diarrhoeal disease. However being able to access source of drinking water within 30 minutes on foot from residents´ household, washing hands with soap or ash and water before eating and after using the toilet, and two or more households sharing a toilet facility were significantly associated with diarrhoeal disease. On multivariate analysis, washing hands with soap or ash and water before eating [AOR = 0.23 (95% CI 0.06-0.90)], washing hands with soap or ash and water after using the toilet [0.16 (95% CI 0.04-0.55)] and two or more households sharing a toilet facility [4.78 (95% CI 2.03-11.24)] remained significantly associated with diarrhoeal disease.

**Table 3:** factors associated with diarrhoeal disease among community residents, Obukpa, Enugu, South East Nigeria, May - June, 2017

Variable	Diarrhoea Yes, n (%)	Diarrhoea No, n (%)	Crude OR (95% CI)	p value	Adjusted OR (95% CI)	p value
**Age (Years)**						
< 5	7 (10.77)	58 (89.23)	1.59 (0.67-3.76)	0.28		
≥ 5	33 (7.04)	436 (92.96)				
**Sex**						
Male	20 (9.17)	198 (90.83)	1.49 (0.78-2.85)	0.22		
Female	20 (6.33)	296 (93.67)				
**Educational status**						
No formal education	4 (4.71)	81 (95.29)	0.60 (0.21-1.77)	0.35		
Formal education	29 (7.55)	355 (92.45)				
**Source of drinking water**						
Improved source	1 (1.52)	65 (98.48)	5.61 (0.75 -41.75)	0.06	6.6 (0.73-57.90)	0.09
Unimproved source	32 (7.94)	371 (92.06)				
**Procurement of water**						
Commercially procured	28 (8.02)	65 (98.48)	2.00 (0.76-5.32)	0.15		
Non-commercially procured	5 (4.17)	115 (95.83)				
**Accessibility to source of drinking water (on foot)**						
≤ 30 minutes	9 (4.37)	197 (95.63)	0.45 (0.21-1.00)	0.05	0.40 (0.27-1.37)	0.23
> 30 minutes	24 (9.13)	239 (90.87)				
**Hand washing before eating**						
Yes	29 (6.36)	427 (93.64)	0.15 (0.04-0.53)	< 0.001	0.23 (0.06-0.90)	0.04
No	4 (30.77)	9 (69.23)				
**Hand washing after using toilet**						
Yes	28 (6.15)	427 (93.85)	0.12 (0.04-0.38)	< 0.001	0.16 (0.04-0.55)	< 0.01
No	5 (35.71)	9 (64.29)				
**Sewage disposal method**						
Open defecation	17 (6.18)	258 (93.82)	0.73 (0.36-1.49)	0.39		
Sanitary method	16 (8.25)	178 (91.75)				
**Number of households sharing toilet facility**						
< 2	22 (5.58)	372 (94.42)	2.91 (1.34-6.28)	0.005	4.78 (2.03-11.24)	< 0.001
≥ 2	11 (14.67)	64 (85.33)				

## Discussion

This study determined the prevalence of diarrhoea and assessed the water, sanitation and hygiene risk factors for diarrhoeal disease in a rural community in South East Nigeria. Overall, the prevalence of diarrhoea in the community studied was 7.49% among all ages and 10.77% among children younger than five years. The overall prevalence of diarrhoea observed in this study was lower than the diarrhoea prevalence rates of between 8.0% and 21.0% reported among the general population from some sub-Saharan African countries [11] but similar to the prevalence rates of between 6.4% and 7.6% reported from some developed countries [12]. The low diarrhoea prevalence rate in the general population observed in this study may be due to seasonal variations in the occurrence of diarrhoea and also possibly due to the high prevalence of hand washing observed among community residents in this study. Children younger than five years had a higher prevalence of diarrhoea in this study. This finding is similar to the 10.0% and 10.8% prevalence of diarrhoea reported for children within this age group in Nigeria and in rural settings in Nigeria respectively [13]. Although a slightly lower prevalence of 9.0% has been reported by a study in Nigeria [14], higher prevalence of diarrhoea among children younger than five years have been reported by other researchers in Nigeria [15, 16] and studies in another African country [17, 18]. Diarrhoea morbidity and mortality has been noted to be higher in children younger than five years because of the combined effects of malnutrition and childhood infections especially in LMICs [1].

Although majority of the community residents sourced drinking water from boreholes, an improved source of water; many procured water commercially from water vendors. Furthermore, many residents had to walk long distances to get to the water collection point thereby possibly exposing water collected to potential contamination by pathogenic causes of diarrhoeal disease. Access to an improved source of water does not guarantee safety of water at the point of consumption. The World Health Organization (WHO) has classified an improved source of water requiring more than a 30-minute round trip for collecting water as an unimproved source [6]. A disproportionately higher number of people in low resource settings such as sub-Saharan Africa still drink water that is, at least occasionally, contaminated with faecal indicator bacteria [6].

The high prevalence of open defecation in this study suggests inadequate access to improved sanitary methods of excreta disposal in the community. This potentially poses the danger of contamination of water sources especially surface water with a resultant increased risk of diarrhoeal disease. Many rural communities in low resource areas including Nigeria lack access to improved sanitation methods with mortality due to inadequate sanitation in Nigeria estimated to be over 27,000 annually [6]. Available evidence suggests that up to 95% of diarrhoeal deaths in children could be prevented by the year 2025 by improving access to safe and accessible excreta disposal amongst other cost effective interventions [19].

This study found high proportions of community residents practicing hand washing with soap or ash and water before eating and after using the toilet. This prevalence of hand washing is five times higher than the reported global prevalence of 19% [6, 20]. The reason for the high prevalence of hand washing observed in this study is unclear but may be due to increased community awareness of the benefits of hand washing with soap and water following nationwide awareness campaigns during the recent outbreak of Ebola Virus Disease (EVD) in Nigeria [21, 22] and also the availability of improved sources of water to most of the households.

In this study, children younger than five years had 59% higher odds of diarrhoeal disease compared to the rest of the population in the community. This was however found not to be statistically significant. This might have been due to the small number of children younger than 5 years in the community studied. Children younger than five years have been reported to have a higher risk of diarrhoeal disease morbidity and mortality [1, 6]. Malnutrition, unsafe water and unsafe sanitation are the leading risk factors for diarrhoeal death among children younger than five years [1, 23].

Hand washing with soap or ash and water before eating and after using the toilet was found to be protective against diarrhoeal disease in this study. Community residents who practiced hand washing before eating and after visiting the toilet had seven and eight times lower odds of diarrhoeal disease respectively compared to those who did not practice hand washing. The findings of this study are similar to the findings from other studies which have reported hand washing at critical moments to be protective against diarrhoeal disease [9, 16, 24-27]. Unsafe water, sanitation and hygiene are still important risk factors for diarrhoeal disease morbidity and mortality in LMICs including sub-Saharan African countries [1, 28]. Interventions which promote hand washing have been shown to lower the risk of diarrhoea morbidity by about 30% [29, 30]. Also basic hygiene practices such as hand washing by care givers could reduce diarrhoeal mortality in children younger than five years [31]. Sharing of toilet facility by two or more households was a risk factor for diarrhoeal disease in this study. Residents of households who shared toilet facility with one or more households had three times the odds of diarrhoeal disease compared to those who do not share toilet facility. Sanitation facilities that are shared or that are unimproved do not ensure adequate and hygienic separation of human excreta from human contact and hence increase the risk of diarrhoeal disease [6].

## Conclusion

The prevalence of diarrhoea was higher among children younger than five years in the communities studied. Sharing of a toilet facility by two or more households was a risk factor for diarrhoea while washing hands with soap or ash and water before eating and after using the toilet protected again diarrhoea. Interventions aimed at increasing access to improved sanitary methods for sewage disposal and ensuring residents of rural communities practice hand washing with soap and water at critical moments would help reduce diarrhoeal disease.

### What is known about this topic

- Diarrhoea morbidity and mortality disproportionately affects developing regions and low resource settings compared to developed regions and high resource settings of the world;
- Risk of diarrhoea morbidity and mortality is higher among children younger than five years;
- Availability and accessibility to water, sanitation and hygiene facilities play important roles in diarrhoeal morbidity.

### What this study adds

- This study highlights the role of hand washing at critical moments on the prevalence of diarrhoea in a rural community;
- The findngs of this study provide evidence supporting hand washing at critical moments as an important factor in preventing diarrhoeal morbidity;
- The findings from this study suggests that to control diarrhoeal morbidity at community level, promoting hand washing practices and improving access to improved sanitation facilities remain important approaches.
